# A Missense Mutation in the *KLF7* Gene Is a Potential Candidate Variant for Congenital Deafness in Australian Stumpy Tail Cattle Dogs

**DOI:** 10.3390/genes12040467

**Published:** 2021-03-24

**Authors:** Fangzheng Xu, Shuwen Shan, Susan Sommerlad, Jennifer M. Seddon, Bertram Brenig

**Affiliations:** 1 Institute of Veterinary Medicine, University of Goettingen, 37077 Göttingen, Germany; fangzheng.xu@stud.uni-goettingen.de (F.X.); shuwen.shan@stud.uni-goettingen.de (S.S.); 2 School of Veterinary Science, The University of Queensland, Gatton, QLD 4343, Australia; s.sommerlad@uq.edu.au (S.S.); j.seddon1@uq.edu.au (J.M.S.)

**Keywords:** deafness, kruppel-like factor 7, genome wide association study, Australian stumpy tail cattle dog, brainstem auditory evoked response

## Abstract

Congenital deafness is prevalent among modern dog breeds, including Australian Stumpy Tail Cattle Dogs (ASCD). However, in ASCD, no causative gene has been identified so far. Therefore, we performed a genome-wide association study (GWAS) and whole genome sequencing (WGS) of affected and normal individuals. For GWAS, 3 bilateral deaf ASCDs, 43 herding dogs, and one unaffected ASCD were used, resulting in 13 significantly associated loci on 6 chromosomes, i.e., CFA3, 8, 17, 23, 28, and 37. CFA37 harbored a region with the most significant association (−log_10_(9.54 × 10^−21^) = 20.02) as well as 7 of the 13 associated loci. For whole genome sequencing, the same three affected ASCDs and one unaffected ASCD were used. The WGS data were compared with 722 canine controls and filtered for protein coding and non-synonymous variants, resulting in four missense variants present only in the affected dogs. Using effect prediction tools, two variants remained with predicted deleterious effects within the Heart development protein with EGF like domains 1 (*HEG1*) gene (NC_006615.3: g.28028412G>C; XP_022269716.1: p.His531Asp) and Kruppel-like factor 7 (*KLF7*) gene (NC_006619.3: g.15562684G>A; XP_022270984.1: p.Leu173Phe). Due to its function as a regulator in heart and vessel formation and cardiovascular development, *HEG1* was excluded as a candidate gene. On the other hand, *KLF7* plays a crucial role in the nervous system, is expressed in the otic placode, and is reported to be involved in inner ear development. 55 additional ASCD samples (28 deaf and 27 normal hearing dogs) were genotyped for the *KLF7* variant, and the variant remained significantly associated with deafness in ASCD (*p* = 0.014). Furthermore, 24 dogs with heterozygous or homozygous mutations were detected, including 18 deaf dogs. The penetrance was calculated to be 0.75, which is in agreement with previous reports. In conclusion, *KLF7* is a promising candidate gene causative for ASCD deafness.

## 1. Introduction

Deafness can cause several inconveniences for dogs (*Canis familiaris*, CFA), as more attention is required to avoid undetected danger. Deaf dogs are not suitable as working dogs because their training is more challenging than for normal hearing dogs. In addition, they are more likely to be startled and show more tendency to bite [1]. More than 100 modern dog breeds have been reported to be affected by congenital deafness [2]. Hence, deafness seems to be a common disorder among dogs, particularly in breeds such as the Dalmatian, Bull Terrier, English Setter, English Cocker Spaniel, and Australian Cattle Dog [3]. Hearing loss or deafness can be categorized mainly by five criteria in dogs: (1) Cause (genetic or nongenetic, inherited or acquired); (2) association with other diseases or phenotypes (syndromic or non-syndromic); (3) number of affected ears (unilateral or bilateral); (4) degree of loss (partial or total); and (5) site of pathology (peripheral or central) [4]. Peripheral deafness can also be classified as inherited or acquired, congenital or late onset, and sensorineural or conductive. In dogs, three classifications of deafness are commonly seen, including inherited congenital sensorineural, acquired later-onset sensorineural, and acquired later-onset conductive deafness [5].

In dogs, congenital sensorineural deafness is common, resulting in total deafness in young puppies that is either unilateral or bilateral. Sensorineural deafness results from dysfunction of cochlea or spiral ganglion. While it can be a degenerative process that relates to aging, noise trauma, exposure to therapeutic drugs that have ototoxic side effects, and chronic conditions [6], it is frequently inherited and so linked to one or more genetic mutations. Some morphological studies in dogs showed congenital sensorineural deafness manifested hypoplasia or aplasia of the sensory cells in the organ of Corti, stria vascularis, macula saccule, solidification, and calcification of tectorial membrane [7,8]. Congenital sensorineural deafness is usually, but not always, related to pigmentation genes in some breeds [3].

Diagnosis of canine deafness usually consists of behavioral or electrodiagnostic testing. The behavioral testing is often unreliable, especially for the unilateral deafness or partial hearing impairment cases. The response of dogs may be affected by psychology (e.g., anxiety or loss of interest) and other senses (e.g., visual cues, vibration, or even air movement) [9]. The brainstem auditory evoked response (BAER) is the averaged record of the electrical activity of the auditory pathway in response to externally applied acoustic stimuli [10]. Compared with behavioral testing, the BAER test is an objective diagnostic method, with the advantages of being easy to record, noninvasive, safe, short test time, and giving reliable results [11].

The Australian Stumpy Tail Cattle Dog (ASCD) is a unique breed with a natural bob-tail, which should be distinguished from the Australian Cattle Dog breed. ASCD is alert, watchful and obedient, and talented in working and controlling cattle. It has been recognized as a standardized breed since 1963 by the Australian National Kennel Council. For a long time, general opinion held that the origins of the Australian Stumpy Tail Cattle Dog arose from European herding dogs and the Australian Dingo. However, recently it has been suggested that the ancestors of the Australian Stumpy Tail Cattle Dog and the Australian Cattle Dog, sharing a common origin, arrived in Australia with early free settlers, as their unidentified companions, between 1788 and c. 1800 (Clark, Noreen R. A Dog for the Job. (in prep. 2020)). Each pup should undergo a BAER test because this breed has a high deafness prevalence (https://www.akc.org/dog-breeds/australian-stump-tail-cattle-dog/ (accessed on 24 March 2021)). A research study of 315 ASCDs showed the incidence of congenital sensorineural deafness was 17.8% [12]. There was no evidence that congenital sensorineural deafness in ASCD has a left/right asymmetry or a sex-specific pattern, but there was a significant correlation between red (over blue) coat color and deafness [12].

No unique causative variants have been identified so far for any dog breeds, possibly in part due to the fact that deafness appears to be a comparatively heterogenous disease as described above. In addition, there are several hypotheses about the inheritance pattern of congenital sensorineural deafness (reviewed by [1]). In Border Collies, for instance, Ubiquitin Specific Peptidase 31 (*USP31*) and RB Binding Protein 6 (*RBBP6*) have been associated with adult-onset deafness [13], whereas in the Doberman Pinscher, an insertion in Protein Tyrosine Phosphatase Receptor Type Q (*PTPRQ*) and a missense variant in Myosin VIIA (*MYO7A*) have been shown to be causative for a form of deafness that includes vestibular disease [14,15]. Although chromosome 2 (CFA2), 6, 14, 17, 27, and 29 have been associated with hearing loss in Dalmatians, no causative variants have been identified so far [16].

In ASCD, congenital sensorineural deafness has been linked to a chromosomal region on CFA10 [12]. However, within a potential candidate gene Sry-related Hmg-box gene 10 (*SOX10*) located in this region, no causative alterations were detected. A recent genome-wide association study (GWAS) reported 14 chromosomes that were significantly associated with deafness in three canine breeds, and CFA3 was significantly associated with bilateral deafness in Australian Cattle Dogs [17]. In this study, three suggestive candidate genes near significantly associated regions were detected in these three dog breeds, including ATPase Na^+^/K^+^ Transporting Subunit Alpha 4 (*ATP1A4*), Transformation/Transcription Domain Associated Protein (*TRRAP*), and Potassium Inwardly Rectifying Channel Subfamily J Member 10 (*KCNJ10*) [17]. However, none have been convincingly identified as causative mutations.

To extend the identification of potential candidate genes causing deafness in ASCD we performed a genome-wide association study and whole genome sequencing (WGS) in deaf ASCD. We identified a unique missense variant in Kruppel-like factor 7 (*KLF7*) gene significantly associated with deafness in ASCDs. This variant was absent in 722 dogs of bioproject PRJN448733 (see below). As *KLF7* plays an important role in the nervous system, is expressed in the inner ear, and seems to be involved in inner ear development [18,19], it was a convincing candidate for ASCD deafness.

## 2. Materials and Methods

### 2.1. Ethical Statement

The collection of dog blood samples was done by S. Sommerlad at the time of BAER testing. The collection of samples was approved by the ‘‘Niedersächsisches Landesamt für Verbraucherschutz und Lebensmittelsicherheit” (33.19-42502-05-15A506) according to §8a Abs. 1 Nr. 2 of the TierSchG. All ASCDs were tested and sampled under approval of The University of Queensland’s Animal Ethics Committee.

### 2.2. Phenotyping and Samples

Fifty-nine Australian Stumpy Tail Cattle Dogs (Table S1) from a previous study [12] were used in this study. BAER testing was performed on 59 dogs [20], 28 were normal hearing dogs and 31 were diagnosed as deaf, of which 10 were bilateral deaf, 12 were left-sided deaf, and 9 were right-sided deaf (Table S1). Three bilaterally deaf ASCDs (#217, #253 and #330), and one control dog with normal hearing (#326) were used for next generation sequencing. Dog #326 was a littermate of #330. These four dogs were female and red in color; all but #330 had a speckled coat. DNA was extracted using a salting-out method as described [12]. All samples were pseudonymized using internal IDs. Furthermore, data from two repository were used in this study. One repository contain Variant Call Format (VCF) data of 722 canine individuals (https://www.ncbi.nlm.nih.gov/bioproject/PRJNA448733 (accessed on 24 March 2021)) [21]. It consists of 144 established breeds, 11 samples with mixed breed, 26 samples with unknown breed status, 104 village and feral dogs from different regions, and 54 wild canids from six species. An additional dataset consisted of 590 samples including 582 dogs from 126 breeds and 8 wolves (https://www.ebi.ac.uk/ena/data/view/PRJEB32865 (accessed on 24 March 2021)) [22].

### 2.3. Next Generation Sequencing and Variant Calling

A total of 1.0 μg DNA per ASCD sample was used as input material for the DNA library preparations. Sequencing libraries were generated using NEBNext^®^ DNA Library Prep Kit following manufacturer’s recommendations and indices were added to each sample. The genomic DNA was randomly fragmented to a size of 350bp by shearing, then DNA fragments were end polished, A-tailed, and ligated with the NEBNext adapter for Illumina sequencing, and further PCR enriched by P5 and indexed P7 oligos. The PCR products were purified (AMPure XP system) and resulting libraries were analyzed for size distribution by Agilent 2100 Bioanalyzer and quantified using real-time PCR. For #217, #253, #326, #330, a total of 599,770,692, 723,624,660, 743,641,356, 620,101,998 raw reads were obtained, respectively. Corresponding coverages were around 40× (paired-end reads, 2 × 150 bp).

Raw sequence data were aligned to dog genome CanFam3.1 using BWA 0.7.17 [23]. SAMtools 1.9 were used for format change and sorting of sequences [24]. Duplicates were marked by PICARD (http://broadinstitute.github.io/picard/ (accessed on 24 March 2021)). Variant calling was performed by GATK 4.1.3 with best practice pipeline [25].

### 2.4. Genome Wide Association Analysis

We used the VCF data obtained in the previous step for GWAS analysis. Three deaf dogs (#217, #253, #330) were used as cases. As ASCD is utilized for control and herding of cattle according to its breed standard (http://www.fci.be/Nomenclature/Standards/351g01-en.pdf (accessed on 24 March 2021)), VCFs of 43 herding dogs from 15 breeds (Australian Cattle Dog, Bearded Collie, Belgian Malinois, Belgian Sheepdog, Belgian Tervuren, Berger Blanc Suisse, Berger Picard, Border Collie, Bouvier des Flandres, Entlebucher Sennenhund, Finnish Lapphund, German Shepherd Dog, Pembroke Welsh Corgi, Shetland Sheepdog, Spanish Water Dog) were extracted from the publicly available 722 canine VCF repository (https://www.ncbi.nlm.nih.gov/bioproject/PRJNA448733 (accessed on 24 March 2021)) [21]. Sample selection criteria were the same as described [21]. A total of 43 herding dogs and the normal hearing dog #326 were chosen as controls (Table S2). The VCF files of the 43 herding dogs and 4 ASCDs were merged by BCFtools 1.9 [24]. Filtering was done using VCFtools 0.1.13 with options --max-alleles 2, --min-alleles 2, --min-meanDP 20, --minQ 20, --minGQ 20, --remove-indels, --max-missing 0.95, --maf 0.05, --hwe 0.001 [26]. After filtering, 857,343 variants remained and were further pruned by Linkage Disequilibrium with –indep 1000 3 1 function in PLINK 1.90 [27]. The final data set consisted of 20,656 single nucleotide polymorphisms (SNPs). Principal component analysis (PCA) was performed using EIGENSOFT package [28]. GEMMA 0.98 was used for association analysis by case–control setting (3 deaf cases vs. one normal hearing ASCD and 43 herding dogs as controls) [29]. A univariate linear mixed model with sex, 5 principal components, and relatedness of 47 dog individuals for corrections was applied for the association test. Bonferroni threshold −log_10_P (0.01/20,656) = 6.32 was utilized. Qqman package was used to generate Manhattan and quantile–quantile (QQ) plots [30]. The genomic inflation factor lambda was calculated with formula lambda = median (qchisq(1-p, 1))/qchisq(0.5, 1) where *p* is a vector of *p* values.

### 2.5. Next Generation Sequencing Data Analysis for Identification of Associated Variants

Data after variant calling were analyzed with SNP & Variation Suite 8.8.3 (Golden Helix Inc., Bozeman, MT, USA). SNPs and indels were set to missing with read depth ≤ 10, genotype quality ≤ 15, alt read ratios for Ref_Ref ≥ 0.15, Ref_Alt outside 0.3 to 0.7, Alt_Alt ≤ 0.85. Variants were analyzed using autosomal recessive and dominant models, respectively. In the autosomal recessive filtering model, 3 deaf ASCDs were set as Alt_Alt, control ASCD as Ref_Ref or Alt_Ref. In the autosomal dominant filtering model, the 3 deaf ASCDs were set as Alt_Alt or Alt_Ref and controls as Ref_Ref. To further narrow the range of candidate variants, we compared the common variants of deaf ASCDs with 722 canine genomes to identify private variants. The shared variants in the three deaf dogs were filtered by BCFtools 1.9 with ‘isec’ option. Private variants were annotated using SnpEFF software [31] to determine high (loss of function) and moderate (missense) impact variants (Ensembl transcripts release 101). These functional variants were further checked by Integrative Genome Viewer (IGV) software to obtain real high quality variants [32]. Variant effects were predicted by SIFT [33], PolyPhen-2 [34], and PROVEAN [35].

### 2.6. Genotyping of KLF7 Variant in Australian Stumpy Tail Cattle Dogs

Targeted genotyping of the KLF7 missense variant was performed in 59 ASCDs by PCR amplification using primers cfa_KLF7_Ex3_F (5′-AGACTCTCTCAGCCGTGGAT-3′) and cfa_KLF7_Ex3_R (5′-GGCCAACTTGTACCACTACCT-3′), resulting in a 295 bp fragment. Genotyping of PCR products were implemented by RFLP analysis after cleavage with the restriction enzyme HinP1I (NEB). The wild type allele was cleaved into two fragments, 236 bp and 59 bp, while the homozygous mutant remained uncut. Frequency distribution for alleles and genotypes was calculated using Fisher’s Exact Test in these 59 ASCDs. Allelic and genotypic odds ratios were calculated according to [36].

### 2.7. Investigation of Human Deafness Genes in 3 Deaf Australian Stumpy Tail Cattle Dogs

Human hearing loss or deafness genes were queried using online software GLAD4U with “hearing loss” and/or “deafness” as keywords [37]. After combining the three query results, 346 genes were chosen for further analysis (Table S3). The variants of these gene regions (including 1000 bp up- and downstream regions) were extracted by BCFtools from VCF files of the three deaf ASCDs and annotated by SnpEFF software. Variants with high (loss of function) and moderate (missense) impacts were selected for further analysis (Ensembl transcripts release 101). The genotype information of the chosen variants was further checked in 722 canines.

## 3. Results

### 3.1. Genome Wide Association Analysis

The analysis was done using three bilateral deaf female dogs from three different litters. The hearing status of the individuals determined using BAER is shown in Table 1 and Table S1.

**Figure 1:**
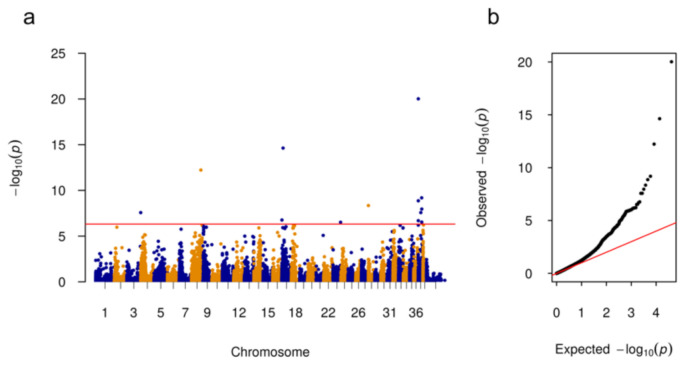
Manhattan and quantile–quantile (QQ) plots illustrating deafness associated chromosomal regions. (**a**) The Manhattan plot shows on the *y*-axis the negative log-base-10 of the *p* value for each of the polymorphisms in the genome (along the *x*-axis), when tested for differences in frequency between 3 bilateral deaf dogs (cases) and 44 controls (1 × ASCD, 43 herding dogs of 15 dog breeds). The red line indicates the Bonferroni significance threshold (−log_10_(0.01/20,656) = 6.32). (**b**) The QQ plot depicts the distribution of *p*-values of the genome-wide association study (GWAS) analysis and genomic inflation factor lambda is 1.20.

The three affected ASCDs were compared with 44 control dogs. 13 SNPs on 6 chromosomes (CFA3, 8, 17, 23, 28, 37) above the Bonferroni significance level were identified. The QQ-plot indicated that some associations might be due to population substructure. Associated SNPs are shown in Figure 1 and summarized in Table 2. The majority of the significantly associated SNPs (7/13) were located on CFA37 including SNP chr37:44793 (position according to CanFam3.1) with the highest −log_10_*p*-value = 20.02. A search for large structural variants (SVs) flanking the significantly associated regions on CFA3, 8, 17, 23, 28, and 37 using IGV was unsuccessful.

### 3.2. Whole Genome Sequencing Reveals Four Potential Variants

To further locate the candidate variants, next generation sequencing was performed in 3 deaf ASCDs (#217, #253, #330) and 1 normal hearing ASCD (#326). After quality control, a total of 4,208,002 SNPs and 2,298,760 indels were detected. According to previous deafness studies, sequence data were initially analyzed using a recessive model of inheritance. Using this model, 129,383 SNPs and 51,942 indels were detected. Using only variants that had been annotated and verified as mRNA transcripts (Ensembl release 101), 338 SNPs and 523 indels remained (Table S4). After filtering these variants against the 722 dog database, none of the homozygous Alt_Alt genotypes were exclusively present in the deaf ASCDs (Table S4). As there were no reports about such a high prevalence of deafness in the 722 control dogs and it can be assumed that the majority of the controls were hearing, these variants were presumably not causative.

As no associated variants were found using the recessive inheritance model, a dominant inheritance model was applied. In this analysis, private variants only present in the three deaf ASCDs (Alt_Alt and Alt_Ref) compared to the 722 controls (Ref_Ref) were filtered, resulting in 270,980 SNPs and 351,927 indels. After quality control and functional annotating, 167 protein-changing variants (58 SNPs and 109 indels) remained (Table S5). These variants were further filtered against #326 (normal hearing littermate of #330) assuming that this dog should be homozygous wild type under the supposed model. After this step, four missense variants remained as potential causative candidates (Table 3). Within the 722 control dogs, no homozygous Alt_Alt or heterozygous carriers were detected for these 4 missense variants. In an additional dataset consisting of 590 dog samples, only two heterozygous individuals (Brussels Griffon dogs) were determined for the Microtubule associated protein 6 (*MAP6*) gene variant. To deduce which of the variants could be causative for deafness, protein function prediction tools were used. As shown in Table 4 only the variants in Heart development protein with EGF like domains 1 (*HEG1*) and *KLF7* were predicted to be deleterious by at least two of the prediction tools.

**Figure 2:**
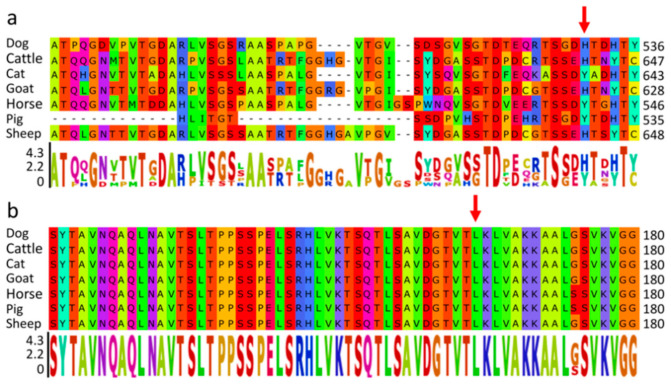
Cross-species comparison of variant amino acid positions in HEG1 and KLF7. Partial protein sequences of HEG1 (**a**) and KLF7 (**b**) flanking the variant amino acid positions were aligned using ClustalW (https://www.ebi.ac.uk/Tools/msa/clustalo/ (accessed on 24 March 2021)). The variant positions are highlighted with a red arrow. Residual color scheme was referred from [38], sequence logos are shown according to [39].

To further confirm the causative possibilities of the two remaining variants, their amino acid conservation was analyzed in the same 7 species. The missense variant in *HEG1* gene (NC_006615.3: g.28028412G>C) resulted in an amino acid exchange of p.His531Asp (XP_022269716.1). In *KLF7* gene (NC_006619.3: g.15562684G>A), the variant led to an exchange of p.Leu173Phe (XP_022270984.1). Especially in KFL7, the amino acid position seems to be highly conserved across several different species, as shown in Figure 2.

### 3.3. Genotyping of KLF7 Variant in ASCDs

To verify the association of the *KLF7* variant with ASCD congenital deafness, 27 normal hearing and 28 deaf ASCDs (21 unilaterally and 7 bilaterally deaf dogs) were used to investigate the *KLF7* variant genotype distribution. As summarized in Table 5, 59 ASCDs including the 4 whole genome sequenced dogs were used to check the association of the *KLF7* missense variant with ASCD deafness. Four dogs were homozygous carriers (A_A) and 14 heterozygous (A_G) among the 31 deaf ASCDs. Within the 28 normal hearing ASCDs, 5 heterozygous and one homozygous carrier were detected. The penetrance of ASCD deafness was calculated to be 0.75. As determined by Fisher’s exact test, homozygosity for the *KLF7* variant was significantly associated with congenital deafness (*p* = 0.014). The odds ratio_AA_ = 6.8 (95% CI [0.68, 67.25]), i.e., homozygous carriers are 6.8 times more likely to be deaf than wild type.

## 4. Discussion

Deafness is a common disorder among dogs, and the observed prevalence is highest in Dalmatians (29.9%) [3] and 17.8% in ASCD [12]. Even selective breeding based on deafness phenotyping decreased the prevalence in Dalmatians only to 17.8% [40]. Several other dog breeds also show rather high prevalence rates (>10%), e.g., Australian Cattle Dog and Bull Terrier [3]. To accelerate the decline of overall prevalence of congenital sensorineural deafness, it would be important to identify the genetic cause of the disorder to enable informed breeding.

We used four ASCD DNA samples from a previous study of deafness in Australian Stumpy Tail Cattle Dogs for GWAS and WGS analysis. The previous study used a genome screen with 325 microsatellite (290 were used for linkage mapping) to determine a significantly linked deafness region on CFA10 [12]. However, *SOX10*, the only potential candidate gene in this region, had to be excluded, as it did not harbor any causative variants. Another promising candidate in the CFA10 region, i.e., Trio- and f-actin-binding protein (*TRIOBP*), had also to be excluded. In the above mentioned study, deafness was reported to be autosomal recessive inherited with incomplete penetrance [12]. As shown before, GWAS with multiple breeds can improve the accuracy of causative variant mapping [41,42]. Our analysis provided evidence for at least six highly associated chromosomal regions. However, due to the small number of affected dogs, some associated regions might have resulted from the close relationship of the dogs. This can be seen in the QQ-plot which showed convincing evidence for an association with some indication of a population substructure. In our study, more than half of the significant associated SNPs (7 out of 13) were located on CFA37, including the most significantly related SNP (chr37:44793, *p* = 9.54 × 10^−^^21^). In a recent study of Dalmatian deafness, signals were also detected in this region [17]. However, there was no associated peak on CFA37 reported in the previous microsatellite-based study in ASCD. A possible explanation could be that there were only five microsatellite markers on CFA37, one of which had a low degree of polymorphism (3 alleles, PIC 0.5) [12]. This might have been insufficient to detect associations on this chromosome. An alternative explanation is that ASCD deafness may be heterogeneous. There may be more than one variant causing congenital deafness in this breed, and using limited family associations may reveal private mutations. Further genotyping analysis in a wider range of affected (28) and unaffected (27) ASCDs revealed that the *KLF7* missense variant was still significantly associated with congenital deafness (Table 5). Furthermore, the penetrance of deafness in ASCD calculated based on the *KLF7* variant was 0.75, which was in agreement with the previously calculated penetrance of 0.72 [12]. Altered allele (A) frequency is 24.58% (Table 5). If we take penetrance into consideration, the deafness frequency is (24.58% × 0.75) = 18.4%, which is also close to the previous investigation of 17.8% overall ASCD breed deafness frequency [12]. Several homozygous wild type individuals were detected among the deaf ASCDs suggesting additional genetic risk factors. This was not surprising, as canine congenital deafness seems to be a complex disorder and different regions were detected in other GWASs for deafness so far [17].

According to our GWAS, functional relationships with deafness of genes near the significantly associated loci on most chromosomes were unapparent (Table 2). Only the region on CFA37 was further supported by WGS. In the initial GWAS 651 variants on chromosome 37 (between CFA37:7217 to CFA37:30803691) were identified (Figure 1). Variant CFA37:15503029T>C with a *p-*value of 8.61 × 10^−^^6^ was only 12,534 bp distant from *KLF7*. To evaluate LD over-pruning and potential effects on resolution, we repeated the GWAS using less stringent pruning parameters (--indep 1000 5 4). This increased the number of associated variants to 60,746. In agreement with the previous analysis, a variant with −log_10_*p*-value = 14.68 at position CFA37:15463045 remained in the vicinity of *KLF7* (Table S6) and a significantly associated region spanning from CFA37:15463045 to CFA37:16433709 was detected harboring *KLF7* (CFA37:15515563-15607345). As expected, a further reduction of pruning stringency resulted in more chromosomal regions above the significant threshold (Figure S1). However, especially on CFA10, no significantly associated variants were identified.

In addition, we applied whole genome sequencing of the deaf dogs and used a large number of available canine whole genome sequence data as controls to improve the accuracy and efficiency of causative variant identification. Several GWAS of canine complex hereditary deafness failed to identify causative variants with the exception of two associated genes (*MYO7A*, *PTPRQ*) causative for a specific form of canine congenital bilateral deafness with vestibular disease [14,15].

For next generation sequence analysis in the present study, functional variants within coding regions were primarily considered due to their direct impact on protein function [43]. We filtered all variants using an autosomal recessive model, however, no functional variants fulfilled this mode of inheritance. Again, the chromosomal region 1 Mb up- and downstream of *SOX10* (CFA10:25680441-27690530) was checked using IGV, but no deafness associated variants including larger structural variants were identified. After WGS analysis and variant effect prediction, only two missense variants within *HEG1* and *KLF7* remained. *HEG1* is involved in cardiovascular development [44] and therefore seemed unlikely to be involved in the development of deafness. However, the candidate variant (NC_006619.3: g.15562684G>A) in *KLF7* (CFA37:15515563-15607345) was close to the significantly associated SNP CFA37:16399127 (*p* = 2.66 × 10^−^^8^) (Table 2). *KLF7* is a zinc finger transcription factor and has been reported to play a role in the nervous system and is vital for neuronal morphogenesis that could function in axon outgrowth [18]. *KLF7* was suggested to have potential functions in neurogenesis of mice, like neuronal differentiation and maturation [45]. *KLF7* was also found to promote axon regeneration [46]. Furthermore, *KLF7* is required for the development of sensory neurons [47], and it has been reported to play roles in neurotransmission and synaptic vesicle trafficking [48]. These two processes have important influences on the auditory system, and therefore disruption of *KLF7* could lead to hearing impairment and dysfunction [49]. Indeed, *KLF7* was confirmed to be expressed in the otic placode which will develop into ears, indicating *KLF7* could have an effect on ear development [19]. *KLF7* was also detected to be a fibroblast growth factor (FGF) responsive factor in ear progenitor induction processes, which implies it may be involved in early ear induction [50]. *KLF7* has been considered as one high quality candidate gene for human branchio-oto-renal syndrome, which is an autosomal dominant disease with hearing loss as one clinical sign [51]. *KLF7* was the nearby gene (50,519 bp distance) of one significant signal in adult hearing difficulty GWAS [52]. One recent GWAS of hearing-related traits with up to 330,759 individuals (UK Biobank) revealed 31 significant genomic risk loci for adult hearing difficulty, *KLF7* was also detected to be significantly associated [53]. Furthermore, the protein sequence segments surrounding KLF7 variant are much more conserved than that of HEG1 among the same 7 species (Figure 2). Recently, KLF7 has been reported to directly regulate GATA Binding Protein 3 (*GATA3*) expression [54]. *GATA3* is expressed in the otic placode and is involved in inner ear development [55]. Though the interaction between *KLF7* and *GATA3* was reported in chicken adipogenesis, KLF7 is quite conserved among several species (Figure 2). Knockdown of Paired Box Protein Pax-2 (*PAX2*) (inner ear development gene) led to a significant up-regulation of both *KLF7* and *GATA3* expression [19], which implies *KLF7* and *GATA3* are probably involved in the same pathway. Furthermore, *GATA3* is the causative gene for human hypoparathyroidism, deafness, and renal dysplasia (HDR) syndrome [56]. Therefore, KLF7 could interact with *GATA3* during the development of inner ear, and defects in KLF7 could affect *GATA3* normal expression patterns in otic placode. This may be a potential cause of hearing loss in ASCD cases. The incomplete penetrance presented by the *KLF7* variant in deafness may be related to its role as a transcription factor that is involved in a specific part of the hearing pathway. Our findings could provide clues for the functional analysis of the *KLF7* in inner ear development. Functional analysis of *KLF7* regarding ear development may provide further evidence for its role in deafness. 

Another intriguing possible pathway is suggested by the finding of a KLF binding site upstream of the M promoter of Microphthalmia-associated transcription factor (*M-MITF*) that induces gene expression changes in humans [57]. Although the aforementioned study was related to melanoma development, *M-MITF* has been identified as the locus responsible for white coat patterning in dogs [58]. Hereditary deafness has been reported to be associated with white pigmentation in several species, e.g., by affecting *M-MITF* isoform expression in pigs [59] and cows [60] as well as humans [61]. Canine deafness was also linked with white pigmentation due to the merle and piebald locus [62]. Congenital sensorineural deafness of English Bull Terrier is predominant in individuals with white coat color [63]. Similarly, congenital hereditary sensorineural deafness in the Australian Cattle Dog was negatively associated with bilateral facial masks, also individuals with pigmented body patches showed a lower risk of deafness [64]. An inverse association of pigmented head patches and congenital sensorineural deafness was also observed in Dalmatians, while on the other hand, a positive correlation was detected with blue irises [65,66,67,68,69,70,71]. In ASCD, congenital sensorineural deafness was moderately significant associated with red/blue coat color, but not with speckling and facial masks [12]. However, no functional alterations in genes related to coat color or pigmentation were detected after filtering for case–control setting in the present study. Thus far, no causative variants within genes involved in pigmentation have been identified in canine deafness. Some pigmentation genes have actually been excluded as candidates in different dog breeds, e.g., c-Kit (*KIT*) and melanocyte protein 17 (*SILV*) [72,73]. An alternative explanation is that deafness caused by dysfunctions of other biological processes may be more common, such as ear development and morphogenesis. This is highly relevant in the Gene Ontology (GO) category analysis of potential canine hereditary deafness genes [2]. In our study, *KLF7* was reported to participate in inner ear development processes [50]. There is good evidence here that the *KLF7* variant contributes to deafness, but the genotyping data supports the view that this is a multigene/multifactorial disease, and so this is one contributing mutation.

## 5. Conclusions

In summary, a missense variant within *KLF7* gene has been identified to be significantly associated with congenital deafness in Australian Stumpy Tail Cattle Dogs. As *KLF7* gene was reported to be expressed in the inner ear and associated with human hearing difficulties, our findings could provide clues for further elucidating novel genetic causes for human hearing loss.

## Figures and Tables

**Table 1:** BAER (brainstem auditory evoked response) results of 4 Australian Stumpy Tail Cattle Dogs (ASCDs).

ID	Gender	Coat Colour	BAER Test Results
217	Female	Red speckled	Bilaterally Deaf
253	Female	Red speckled	Bilaterally Deaf
330	Female	Red	Bilaterally Deaf
326	Female	Red speckled	Normal Hearing

**Table 2:** Significantly associated SNPs above Bonferroni significance threshold (6.32).

CFA	Position	*p*-Value	Nearby Genes	Distance (bp)
3	90,987,932	2.67 × 10^−8^	*LCORL*	186,575
8	62,032,863	5.93 × 10^−13^	*DGLUCY*	19,884
17	1,977,343	1.73 × 10^−7^	*EIPR1*	0
17	9,456,133	2.34 × 10^−15^	*TRIB2*	204,307
23	50,096,314	3.04 × 10^−7^	*KCNAB1*	0
28	21,516	4.50 × 10^−9^	*PTPN20*	42,882
37	13,393	2.04 × 10^−7^	*WDR75*	144,007
37	44,793	9.54 × 10^−21^	*WDR75*	112,607
37	80,438	1.36 × 10^−9^	*WDR75*	76,962
37	16,399,127	2.66 × 10^−8^	*CRYGD*	25,757
37	22,102,392	6.48 × 10^−10^	*ABCA12*	34,340
37	22,579,983	2.93 × 10^−7^	*FN1*	57,573
37	22,711,697	1.10 × 10^−8^	*FN1*	189,287

**Table 3:** Genotype information of four potential causative variants for ASCD deafness.

Chr	HGVS Genome Position ^(a)^	Variant Type	Gene ^(b)^	#217	#253	#330	#326
13	NC_006595.3:g.60805542 C>T	missense variant	*GC*	C_T	C_T	C_T	C_C
21	NC_006603.3:g.23019999 C>T	missense variant	*MAP6*	C_T	C_T	C_T	C_C
33	NC_006615.3:g.28028412 G>C	missense variant	*HEG1*	G_C	G_C	G_C	G_G
37	NC_006619.3:g.15562684 G>A	missense variant	*KLF7*	A_A	A_A	A_G	G_G

^(a)^ Positions according to CanFam3.1; ^(b)^ *GC*: GC vitamin D binding protein, *MAP6*: Microtubule associated protein 6, *HEG1*: Heart development protein with EGF like domains 1, *KLF7*: Kruppel-like factor 7.

**Table 4:** Variant effect predicted by SIFT, PolyPhen-2, and PROVEAN.

Gene	Amino Acid Exchange	SIFT	Polyphen-2	PROVEAN
*GC*	p.Gly389Rrg	Tolerated	Benign	Neutral
*MAP6*	p.Arg486Cys	Affect protein function	Benign	Neutral
*HEG1*	p.His531Asp	Affect protein function	Unknown	Deleterious
*KLF7*	p.Leu173Phe	Affect protein function	Possibly damaging	Neutral

SIFT: https://sift.bii.a-star.edu.sg (accessed on 24 March 2021), Polyphen-2: http://genetics.bwh.harvard.edu/pph2/index.shtml (accessed on 24 March 2021), PROVEAN: http://provean.jcvi.org/index.php (accessed on 24 March 2021).

**Table 5:** Genotype distribution of *KLF7* variant in 31 deaf and 28 normal hearing ASCDs dogs.

Phenotype	G_G	A_G	A_A	Total Number	*P* ^(c)^
Unilaterally deaf	10	10	1	21	0.054
Bilaterally deaf	3	4	3	10	0.010
Deafness (uni ^(a)^ + bi ^(b)^)	13	14	4	31	0.014
Normal hearing	22	5	1	28	

^(a)^ uni: Unilaterally deaf; ^(b)^ bi: Bilaterally deaf; ^(c)^ *p*-value using Fisher’s exact test.

## Data Availability

The data presented in this study are openly available in Center for Open Science (OSF) reference number https://osf.io/z36ap (accessed on 24 March 2021).

## Supplementary Materials

The following are available online at https://www.mdpi.com/2073-4425/12/4/467/s1, Figure S1. Manhattan and QQ plots of the Genome Wide Association Analysis (GWAS) for ASCD deafness. Table S1: *KLF7* variant genotypes of 59 ASCDs. Table S2: Dog breeds of herding group. Table S3: Human deafness related genes. Table S4: Variants of recessive inheritance model and annotated with Ensembl release 101. Table S5: Private variants and annotated with Ensembl release 101. Table S6: Significantly associated SNPs above Bonferroni significance threshold (6.78).
