# Supplementary material for: A Missense Mutation in the KLF7 Gene Is a Potential Candidate Variant for Congenital Deafness in Australian Stumpy Tail Cattle Dogs
Source: Genes (Basel). 2021 Mar 24;12(4):467. doi: 10.3390/genes12040467 (PMC8064056; doi:10.3390/genes12040467)
Supplement: Supplementary file 1 [file genes-12-00467-s001.zip › Supplementary_files/Figure S1.docx]

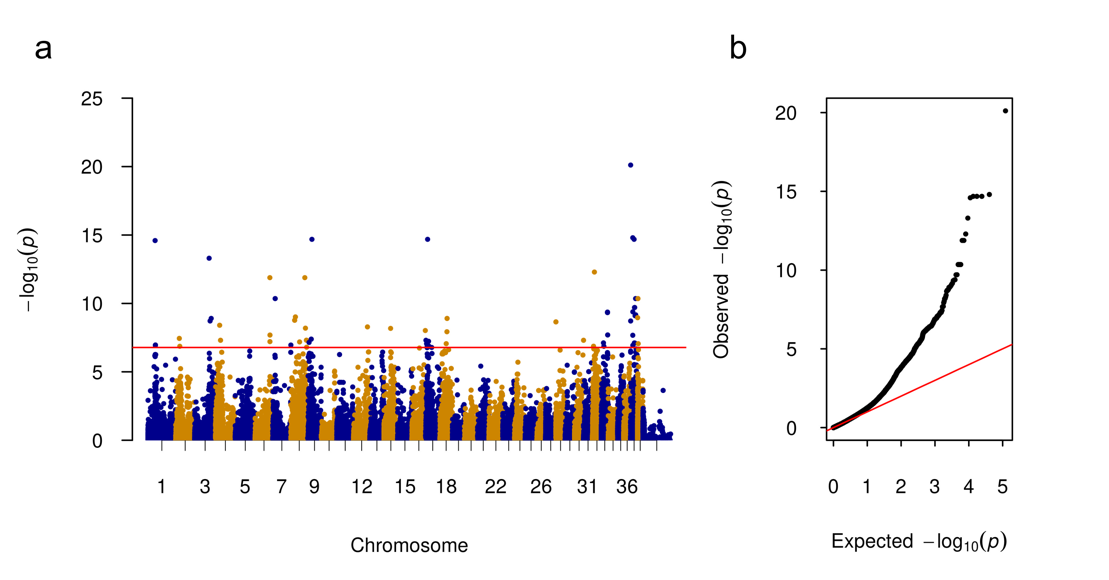


**Figure** **S1**. Manhattan and QQ plots of the Genome Wide Association Analysis (GWAS) for ASCD deafness. **(a)** Association of 60,746 variants with bilateral deafness in 47 dogs (3 cases and 44 controls). The plot shows the -log_10_*p*-values for all variants. 60,746 were remained after pruning by Linkage Disequilibrium with parameters --indep 1000 5 4. The red horizontal line represents the Bonferroni genome-wide significance threshold of −log_10_(0.01/60,746) = 6.78. **(b)** Quantile-quantile (QQ) plot showed the observed -log_10_*p*-values in the black curve, the red line indicated the distribution of expected -log_10_*p*-values. λ is calculated to be 1.10.
