# Supplementary material for: A Missense Mutation in the KLF7 Gene Is a Potential Candidate Variant for Congenital Deafness in Australian Stumpy Tail Cattle Dogs
Source: Genes (Basel). 2021 Mar 24;12(4):467. doi: 10.3390/genes12040467 (PMC8064056; doi:10.3390/genes12040467)
Supplement: Supplementary file 1 [file genes-12-00467-s001.zip › Supplementary_files/Table S6.docx]

**Table S6**. Significantly associated SNPs above Bonferroni significance threshold (6.78).

| **CFA** | **Position** | **P-value** |
| --- | --- | --- |
| 1 | 31233575 | 2.55e-15 |
| 1 | 32395183 | 1.16e-07 |
| 1 | 32475384 | 1.13e-07 |
| 1 | 32736021 | 1.08e-07 |
| 2 | 16435097 | 3.60e-08 |
| 2 | 18543997 | 1.35e-07 |
| 3 | 63628899 | 4.98e-14 |
| 3 | 67878509 | 1.91e-09 |
| 3 | 72889394 | 1.25e-09 |
| 4 | 18752526 | 3.93e-09 |
| 4 | 22681833 | 4.92e-08 |
| 6 | 65044531 | 1.31e-12 |
| 6 | 65403131 | 6.15e-08 |
| 6 | 65581389 | 2.05e-08 |
| 7 | 11106974 | 4.43e-11 |
| 7 | 80963174 | 1.10e-07 |
| 8 | 16659846 | 1.70e-09 |
| 8 | 20053234 | 9.72e-10 |
| 8 | 38701796 | 6.47e-08 |
| 8 | 62032863 | 1.31e-12 |
| 8 | 64801907 | 6.41e-09 |
| 8 | 69175082 | 4.86e-08 |
| 8 | 70060784 | 1.58e-07 |
| 9 | 6623401 | 7.02e-08 |
| 9 | 16257629 | 4.06e-08 |
| 9 | 19247396 | 2.07e-15 |
| 12 | 61700672 | 5.17e-09 |
| 14 | 29191852 | 6.67e-09 |
| 16 | 58797193 | 9.56e-09 |
| 17 | 1977343 | 4.92e-08 |
| 17 | 4806908 | 8.72e-08 |
| 17 | 7576793 | 1.31e-07 |
| 17 | 9456133 | 2.07e-15 |
| 17 | 13630753 | 5.83e-08 |
| 17 | 14473620 | 1.28e-07 |
| 18 | 28211327 | 8.72e-08 |
| 18 | 31369439 | 1.18e-08 |
| 18 | 32020162 | 1.25e-09 |
| 28 | 21516 | 2.23e-09 |
| 30 | 39972993 | 4.92e-08 |
| 32 | 4402731 | 1.34e-07 |
| 32 | 8234087 | 5.07e-13 |
| 33 | 9885601 | 7.60e-08 |
| 33 | 11734473 | 1.35e-07 |
| 33 | 27695032 | 4.71e-10 |
| 33 | 27698649 | 4.31e-10 |
| 33 | 28492490 | 2.02e-08 |
| 37 | 44793 | 7.72e-21 |
| 37 | 80438 | 1.91e-09 |
| 37 | 9475356 | 1.58e-15 |
| 37 | 9488831 | 2.13e-08 |
| 37 | 9730850 | 4.11e-10 |
| 37 | 9791864 | 1.15e-07 |
| 37 | 15463045 | 2.07e-15 |
| 37 | 16433709 | 7.71e-08 |
| 37 | 17097084 | 2.00e-10 |
| 37 | 18171605 | 1.97e-10 |
| 37 | 19121465 | 7.86e-08 |
| 37 | 19744271 | 7.97e-10 |
| 37 | 22102392 | 4.43e-11 |
| 37 | 22711697 | 6.86e-10 |
| 38 | 338354 | 1.11e-09 |
| 38 | 1842394 | 4.43e-11 |
| 38 | 3008104 | 8.71e-08 |
